# The Milton Hospital

**Published:** 1909-11-20

**Authors:** James McGregor


### Editor's Letter-Box.

### THE MILTON HOSPITAL.

*To the Editor of* The Hospital.

Sir,—May I ask you to be good enough to correct the paragraph as to Portsmouth Isolation Hospital appearing in your issue of November 13? The father of the child if employed in the dockyard is compelled to remain at home as long as his child remains in the house only, and not for several days, when his child is sent home convalescent. The temporary building is to be erected for the purpose of dealing with a large number of scarlet fever cases waiting admission. Convalescent wards exist at present.

Yours truly,

James McGregor.

